# Physical Workload and Work Capacity across Occupational Groups

**DOI:** 10.1371/journal.pone.0154073

**Published:** 2016-05-02

**Authors:** Stefanie Brighenti-Zogg, Jonas Mundwiler, Ulla Schüpbach, Thomas Dieterle, David Paul Wolfer, Jörg Daniel Leuppi, David Miedinger

**Affiliations:** 1 University Clinic of Internal Medicine, Cantonal Hospital Baselland, Liestal, Switzerland; 2 Department of Health Sciences and Technology, ETH Zurich, Zurich, Switzerland; 3 Department of Sport Exercise and Health, University of Basel, Basel, Switzerland; 4 Faculty of Medicine, University of Basel, Basel, Switzerland; 5 Institute of Anatomy, University of Zurich, Zurich, Switzerland; 6 Department of Occupational Medicine, Swiss National Accident Insurance Fund, Lucerne, Switzerland; Vanderbilt University, UNITED STATES

## Abstract

This study aimed to determine physical performance criteria of different occupational groups by investigating physical activity and energy expenditure in healthy Swiss employees in real-life workplaces on workdays and non-working days in relation to their aerobic capacity (VO_2max_). In this cross-sectional study, 337 healthy and full-time employed adults were recruited. Participants were classified (nine categories) according to the International Standard Classification of Occupations 1988 and merged into three groups with low-, moderate- and high-intensity occupational activity. Daily steps, energy expenditure, metabolic equivalents and activity at different intensities were measured using the SenseWear Mini armband on seven consecutive days (23 hours/day). VO_2max_ was determined by the 20-meter shuttle run test. Data of 303 subjects were considered for analysis (63% male, mean age: 33 yrs, SD 12), 101 from the low-, 102 from the moderate- and 100 from the high-intensity group. At work, the high-intensity group showed higher energy expenditure, metabolic equivalents, steps and activity at all intensities than the other groups (p<0.001). There were no significant differences in physical activity between the occupational groups on non-working days. VO_2max_ did not differ across groups when stratified for gender. The upper workload limit was 21%, 29% and 44% of VO_2max_ in the low-, moderate- and high-intensity group, respectively. Men had a lower limit than women due to their higher VO_2max_ (26% vs. 37%), when all groups were combined. While this study did confirm that the average workload limit is one third of VO_2max_, it showed that the average is misrepresenting the actual physical work demands of specific occupational groups, and that it does not account for gender-related differences in relative workload. Therefore, clinical practice needs to consider these differences with regard to a safe return to work, particularly for the high-intensity group.

## Introduction

Serious injury or illness can lead to a significant loss of working hours and substantial health care costs, when an employee is no longer able to perform his/her work appropriately. In 2014, about 226,000 people (4% of the insured population) received disability pension in Switzerland amounting to approximately 370 million US Dollars [1]. Adult recipients were predominantly male (53%) and older than 45 years [1]. Main reasons for disability were attributable to illness (79%), with a considerable proportion of musculoskeletal disorders (19%) [1]. Physical workload was found to be an independent risk factor for disability retirement due to musculoskeletal disorders [2]. When reintegrating patients into the work process after phases of sick leave, employers and insurance agencies may have to assess the work capacity of a person in order to adequately adjust the job profile [3]. The knowledge of workload and required work capacity could facilitate this process, because a successful resumption of work is highly dependent on these factors [3]. Since physical workload differs considerably between job assignments, it is mandatory to analyse a wide range of physical work requirements and to assess employees’ work capacity across occupational groups. Although several attempts have been made in this regard, there are few objective data available so far and no established reference values exist to evaluate whether, when and how a return to work is possible [4]. Therefore, this study aimed to describe the relationship between physical workload and work capacity in order to provide the basis for guiding occupational rehabilitation measures.

Regarding workload, the Dictionary of Occupational Titles (DOT) [5] has been developed by the US government in order to classify professions into five categories based on the amount of energy expenditure (EE) as well as on the intensity and duration of lifting or carrying during work. However, the DOT has not been based on quantitative work-related analyses and its validity has not been established [4]. Work capacity can be assessed using functional capacity evaluations (FCEs) [6]. Soer *et al*. [3] applied an evaluation system consisting of 12 work-related tests to establish functional capacity of healthy employees. The assessment included various lifting and energetic exercises as well as coordination tasks. From the test results, normative FCE values were developed for each DOT-category in healthy adults, which could be compared to patient data in order to make return-to-work recommendations [3]. However, since validity of the DOT has not been proved, further analysis concerning workload assessment is required. Previous studies measuring physical activity in employees have used pedometers or accelerometers in combination with self-reported questionnaires [7–9]. Step counting using pedometers or accelerometers is widely accepted for assessing the amount of physical activity [10]. However, an accurate assessment of physical work requirements is not possible using these instruments. The SenseWear Mini armband (SWMA) not only measures step counts but also captures EE with a multi-sensory system based on thermogenic properties [11]. The combination of multiple sensors enables it to overcome limitations of conventional devices. By measuring heat produced by the body, the armband can detect EE associated with load carrying and free-living physical activities [11]. Regarding work capacity, the application of FCE tools is time- and labour-intensive and may therefore not be appropriate in a clinical or field context. In contrast, aerobic capacity or cardiorespiratory fitness as measured as maximal oxygen uptake (VO_2max_) has been shown to be an adequate indicator for assessing individuals’ work capacity [12].

Objective workload data as measured by the SWMA in relation to work capacity as measured by VO_2max_ have not been evaluated so far. Therefore, the primary aim of the present study was to determine detailed activity profiles of different occupational groups by investigating aerobic capacity and physical activity demands on workdays and non-working days in healthy Swiss employees. Furthermore, predictors of physical workload were analysed. As a secondary objective, the International Physical Activity Questionnaire (IPAQ) was co-evaluated in order to allow comparison to a simple assessment tool.

## Materials and Methods

### Study subjects

From May 17 2013 (first participant in) to February 11 2015 (last participant in), 337 healthy and full-time employed adults (≥80% full-time equivalent) from various companies of the Basel region, Switzerland were recruited. Exclusion criteria were insufficient knowledge of the German language, movement restrictions as well as diseases and accidents within the past three months that affected productivity at the workplace. Furthermore, night shift workers could not take part in this study because of their altered sleep, eating and physical activity behaviour. This investigation has been conducted according to the Declaration of Helsinki and was approved by the local ethics committee “Ethikkommission Nordwest- und Zentralschweiz” (EKNZ, 260/12) on December 21 2012. Written informed consent was obtained from all participants prior to study entry.

### Study design and procedures

In this cross-sectional study, the aim was to recruit an equal distribution of subjects across different occupational groups. Based on this, appropriate companies were selected and addressed by a member of the research team, including medium sized corporations from the public sector (e. g. hospitals) as well as small sized private firms (e. g. construction companies). A permit from leading persons was requested to receive contact details for potentially recruitable employees, who were then informed and asked for study participation by phone or by email. At the first study visit, height and weight were reliably measured. Height was assessed without shoes by a medical measuring stick to the nearest mm (model Seca 217, measurement range: 20 to 205 cm, Seca AG, Reinach, Switzerland). The measurement of weight was performed on subjects in light clothing without shoes by a medical scale with an accuracy of 0.1 kg (model Seca 877, load capacity: 200 kg, Seca AG, Reinach, Switzerland). Body mass index (BMI) was calculated from measured height and weight (BMI = weight/height^2^ [kg/m^2^]). Subjects with a BMI of ≥25 kg/m^2^ were classified as overweight, and those with a BMI of ≥30 kg/m^2^ as obese [13]. In addition, various personal and job-related factors were recorded by a generic questionnaire, such as age, gender, nationality, marital status, smoking status, alcohol consumption, highest education, current profession, daily working hours, working time model, medication, psychotherapy, illnesses and accidents within the last three months. Based on the reported professions, subjects were classified (nine categories) according to the International Standard Classification of Occupations 1988 (ISCO-88) [14] and merged into three groups with low- (managers, scientists, office workers), moderate- (technicians, service workers, machine operators) and high-intensity occupational activity (agricultural workers, craftsmen, labourers) [15]. Prior to the observation period, subjects performed a 20-meter shuttle run test in order to measure aerobic capacity. During the subsequent week, participants wore the SWMA on seven consecutive days in order to objectively measure daily physical activity. It was ensured that the examination week consisted of at least three workdays. One week later at the second study visit, subjects completed the self-reported IPAQ.

### Physical activity assessment

#### SenseWear mini armband

The SWMA (model MF-SW) is a small, lightweight and wireless multisensory activity monitor developed by BodyMedia Inc., Pittsburgh, Pennsylvania, USA (now Jawbone Inc., San Francisco, California, USA), which integrates a three-axis accelerometer along with other sensors such as heat flux, skin temperature and galvanic skin response. Validity was established by Johannsen *et al*. [11] comparing EE estimates of the SWMA against the criterion method Doubly-Labeled-Water in healthy adults. Subjects were instructed to wear the SWMA on the upper left arm (triceps area) for seven consecutive days, including while sleeping, with the exception of one hour daily spent on personal hygiene. The first and last incomplete measurement day, including the study visits, were not taken into account. Therefore, the investigated measurement period was five days, which had to consist of at least three workdays to be included in the analysis [16]. A day was considered as a whole workday, if participants worked cumulatively ≥6 hours, and as a half workday in case of ≥3 to <6 hours. Days with <3 working hours were regarded as non-working days. Measurement days of <22 hours per day or <12 hours during wake time were excluded from analysis [17, 18]. Information about workdays and non-working days as well as work-time and leisure-time on workdays was obtained from diaries participants filled in during the measurement period.

The physiological data collected by the armband’s sensors were processed by specific algorithms available in the SWMA software (BodyMedia, professional software V.7.0, algorithm V.2.2.4). Participants’ daily EE, metabolic equivalents (METs), physical activity duration at different intensities and number of steps were calculated. One MET corresponds to 3.5 ml/kg/min VO_2_ [19]. Moderate physical activity (MPA) was defined as 3–6 METs, high physical activity (HPA) as 6–9 METs and very high physical activity (VHPA) as ≥9 METs. For all variables, average values were computed separately for workdays and non-working days as well as work-time and leisure-time on workdays. To have a measure for total recreation, mean values of leisure-time on workdays and non-working days were summed up and divided by the number of analysed days.

#### International physical activity questionnaire

The IPAQ is a simple instrument for measuring physical activity at the population level. Validity and reliability were established in 12 different countries [20]. The German long version of the IPAQ designed for adults aged 15 to 69 years was administered to the participants. It includes 26 questions and assesses past-week frequency and duration of physical activity within the domains of work, leisure-time, transport, domestic and garden. Moreover, each domain consists of walking, moderate and vigorous activities. Continuous scores were calculated for MPA and HPA during work and total recreation. Regarding work, the duration of MPA (min/day) was determined by the sum of walking (3.3 METs) and moderate (4 METs) activity minutes from the work-domain [21]. For recreation, walking and moderate activity minutes from the domains of leisure-time, transport, domestic and garden were added up. To compute the duration of HPA (min/day) during work and recreation, vigorous (8 METs) activity minutes were considered within the corresponding domains [21].

### Evaluation of aerobic capacity: 20-meter shuttle run test

The multistage 20-meter shuttle run is a common endurance fitness test used to evaluate maximal aerobic capacity of healthy adults [22]. It is simple in use, economical and large groups can be tested simultaneously. Validity of the one-minute stage version of the 20-meter shuttle run was established by Léger *et al*. [22], who compared the maximal shuttle run speed to VO_2max_ attained during a multistage treadmill test (r = 0.90). Test-retest reliability was found to be r = 0.95 in healthy adults [23]. This test was conducted on a flat, non-slip surface. Participants were instructed to run back and forth between two lines, which were 20 meters apart, with a running velocity determined by audio signals [23]. Starting speed was 8.5 km/h and every minute (stage), speed was increased by 0.5 km/h until the subject could no longer keep the pace and did not reach the lines in time twice in a row [23]. The test result corresponded to the number of reached stages and shuttles and was used to predict VO_2max_ according to a validated table [24]. Four participants did not perform the 20-meter shuttle run test due to a resting systolic blood pressure >180 mm Hg and were pairwise excluded from the corresponding analyses.

### Determination of physical performance criteria

In order to determine physical performance criteria of different occupational groups, the ratio between workload and employees’ work capacity was analysed. METs during work-time assessed by the SWMA were used as objective measure of workload and VO_2max_ as measure of maximum work capacity. VO_2max_ was converted into METs [19]. To represent 95% of the normal range within each occupational group, workload was expressed as minus (lower limit) and plus (upper limit) two standard deviations (SD) [25]. The lower limit was considered as minimum work requirement for a particular job group. To describe the individual’s work ability in relation to population-based values, the following formula was used: (Individual’s VO_2max_ / Mean VO_2max__Group_x_) x Mean workload_Group_X_.

### Statistical analysis

Data were analysed using the software IBM SPSS Statistics (version 22.0). A p-value of <0.05 was considered as statistically significant. Data are presented as counts and percentages or mean and SD. The Shapiro-Wilk test was used to test whether data were normally distributed. To analyse differences between occupational groups, mean comparisons were performed using One-way Analysis of Variance or Kruskal-Wallis test, if appropriate. Categorical data were analysed with Chi-Square test. Multiple linear regression analyses were performed using the forward stepwise method in order to identify the most important predictors of physical workload. Validity of the regression models was established by checking essential assumptions. EE measured by the SWMA was subject to power calculation. Assuming a sample size of 100 subjects in each occupational group, there is a power of >90% to detect a mean difference of 500 kcal between any of these groups. This calculation was based on the assumption of a within group SD of 730 kcal and on a two-sided significance level of 5% [26].

## Results

### Subjects’ characteristics

Of the 337 recruited subjects 303 were considered for analysis, 101 from the low-, 102 from the moderate- and 100 from the high-intensity group. Age of the analysed participants ranged from 18 to 61 years (mean age: 33 yrs, SD 12) and two-thirds (n = 190, 63%) were male. Mean BMI was 24 kg/m^2^, SD 3, while 31% (n = 95) were found to be overweight and 7% (n = 21) were obese. Further details on study participants are given in S1 Table.

Thirty-four subjects (10%) have worn the SWMA on less than three workdays and were therefore excluded from the entire analysis. Reasons for non-wearing or non-evaluation were: technical problems (n = 4), illness during observation period (n = 2), no paid occupation (n = 5), no interest (n = 15), loss of the armband (n = 4), skin irritations (n = 2) or sleep problems (n = 2). Another 24 individuals had missing SWMA data on non-working days due to more workdays during observation period and were pairwise excluded from the corresponding analyses.

### Classification of occupations

**Fig 1:**
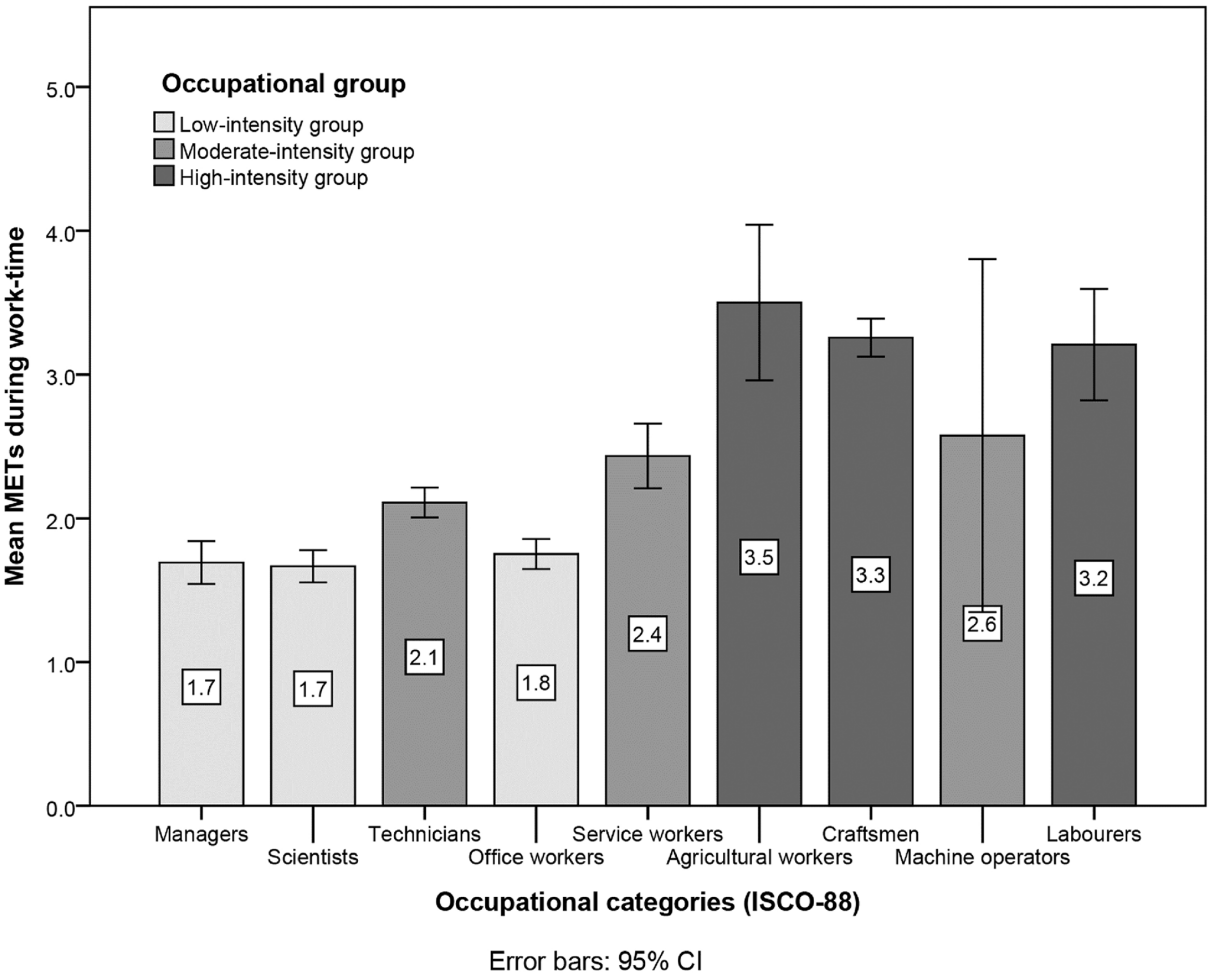
Differences in METs during work-time across occupational categories of the ISCO-88. CI, confidence interval; ISCO-88, International Standard Classification of Occupations 1988; METs, metabolic equivalents. Intergroup comparisons (low-intensity vs. moderate-intensity vs. high-intensity group) revealed highly significant differences (p<0.001).

Looking at METs during work-time across occupational categories (Fig 1), agricultural workers (n = 9), craftsmen (n = 78) and labourers (n = 13) showed significantly higher METs than technicians (n = 74) and service workers (n = 24) (p<0.001) as well as managers (n = 25), scientists (n = 35) and office workers (n = 41) (p<0.001). Technicians and service workers differed significantly from managers, scientists and office workers (p<0.001).

### Physical activity data across occupational groups

Table 1 presents selected demographic characteristics, aerobic capacity and objective SWMA activity parameters across occupational groups. Univariate analyses revealed that the high-intensity group included more males and younger individuals compared to the other groups, whereas BMI did not differ significantly. Furthermore, employees of the high-intensity group showed higher activity levels on workdays (except VHPA). These differences mainly occurred during work-time, while in leisure-time VHPA and steps were reduced in the high-intensity group. In contrast, no significant differences in physical activity were found on non-working days (except EE). Physical activity parameters were generally higher on workdays compared to non-working days in the moderate- and high-intensity group, while it was the contrary in the low-intensity group. Moreover, work-time activity in comparison to leisure-time activity was increased in the high-intensity group, balanced in the moderate- and reduced in the low-intensity group. VO_2max_ was significantly higher in the high-intensity group, but did not differ when stratified for gender (see S2 and S3 Tables for more details). Mean VO_2max_ was 36% higher in men (45 ml/kg/min, SD 8) than in women (33 ml/kg/min, SD 7).

**Table 1:** Aerobic capacity and objective SenseWear activity data across occupational groups (n = 303).

	Low-intensity group (n = 101)		Moderate-intensity group (n = 102)		High-intensity group (n = 100)		
	*N*	*%*	*N*	*%*	*N*	*%*	*p-value*
Males	54	53	41	40	95	95	**<0.001**
	*Mean*	*SD*	*Mean*	*SD*	*Mean*	*SD*	
Age							
[yrs]	38	11	35	12	27	12	**<0.001**
BMI							
[kg/m^2^]	24	3	24	4	25	3	0.121
VO_2max_							
[ml/kg/min]	39	10	38	9	43	8	**<0.001**
EE							
Workday [kcal]	2276	441	2564	852	3563	682	**<0.001**
*Work-time [kcal]*	1050	282	1251	336	2157	461	**<0.001**
*Leisure-time [kcal]*	1227	334	1313	740	1406	455	**0.002**
Non-working day [kcal]	2147	831	1981	544	2333	667	**0.001**
METs							
Workday	2.0	0.3	2.2	0.4	2.8	0.5	**<0.001**
*Work-time*	1.7	0.3	2.2	0.5	3.3	0.6	**<0.001**
*Leisure-time*	2.4	0.4	2.2	0.4	2.3	0.5	0.106
Non-working day	2.1	0.4	2.1	0.5	2.1	0.6	0.868
MPA							
Workday [min]	156	70	215	107	405	135	**<0.001**
*Work-time [min]*	52	42	109	78	294	109	**<0.001**
*Leisure-time [min]*	105	46	107	55	111	49	0.559
Non-working day [min]	174	90	170	103	191	121	0.623
HPA							
Workday [min]	10	10	12	17	28	22	**<0.001**
*Work-time [min]*	1	2	3	7	19	18	**<0.001**
*Leisure-time [min]*	9	10	9	13	9	10	0.780
Non-working day [min]	11	15	10	23	13	17	0.147
VHPA							
Workday [min]	2.3	5.4	2.1	4.2	1.8	5.5	0.339
*Work-time [min]*	0.0	0.1	0.1	1.0	0.4	2.0	**0.001**
*Leisure-time [min]*	2.4	5.5	1.9	4.0	1.5	5.1	**0.040**
Non-working day [min]	2.0	5.9	2.5	7.3	1.9	9.0	0.688
Steps							
Workday	9777	3105	11’674	3661	15’057	4197	**<0.001**
*Work-time*	3650	1760	5824	2514	10’131	3804	**<0.001**
*Leisure-time*	6127	2885	5850	2474	4926	2243	**0.002**
Non-working day	8764	3808	9212	4507	9039	9089	0.181

BMI, body mass index; EE, energy expenditure; METs, metabolic equivalents; MPA / HPA / VHPA, physical activity duration at moderate (3–6 METs) / high (6–9 METs) / very high (≥9 METs) intensity; SD, standard deviation; VO_2max_, maximal oxygen uptake during 20-meter shuttle run test. Significant p-values are highlighted in bold.

**Fig 2:**
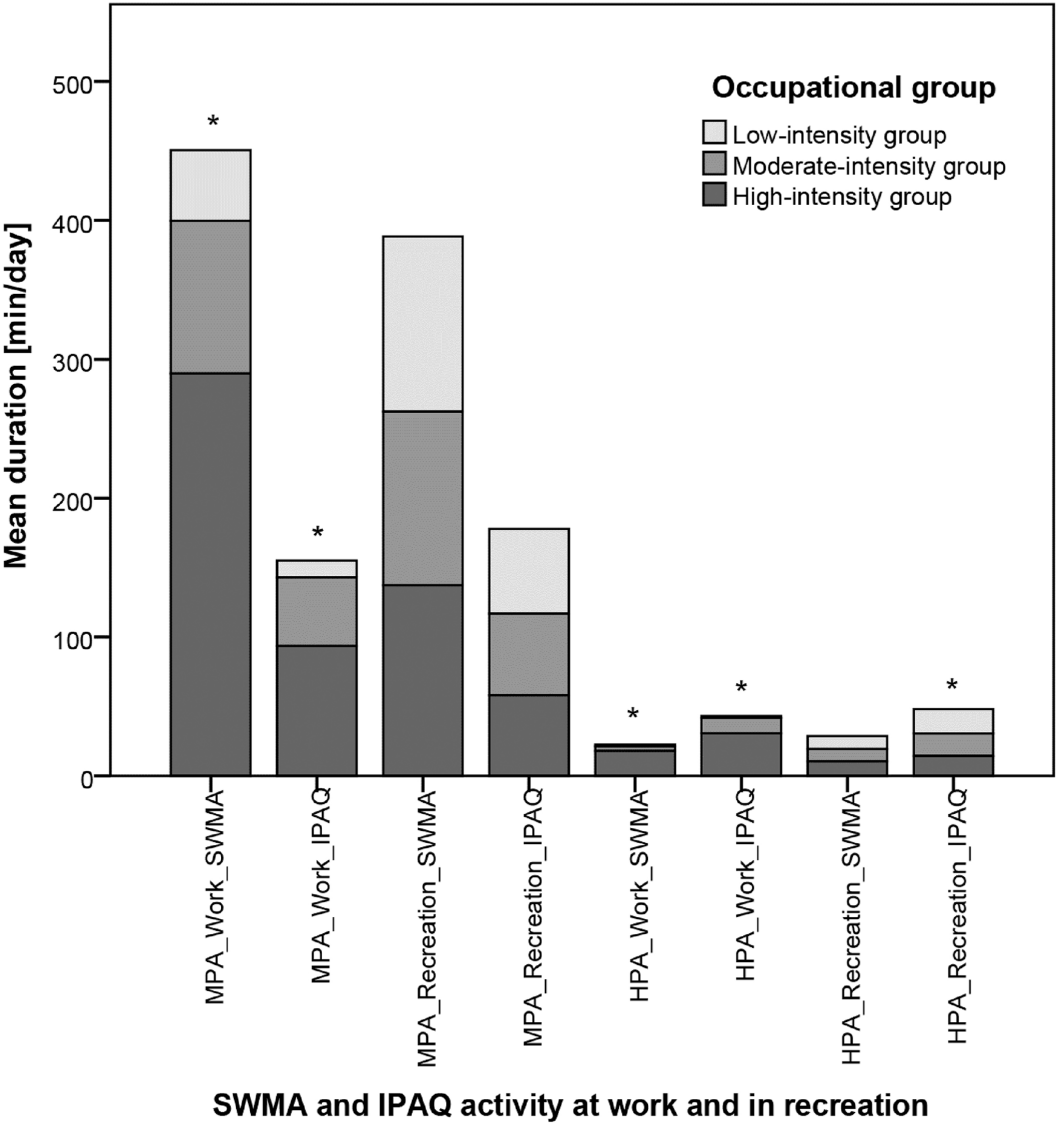
Comparison of objective SWMA activity data with subjective IPAQ activity data. HPA_Recreation_IPAQ / HPA_Work_IPAQ, physical activity duration at high intensity (8 METs) based on the International Physical Activity Questionnaire; HPA_Recreation_SWMA / HPA_Work_SWMA, physical activity duration at high intensity (6–9 METs) measured by the SenseWear Mini armband; MPA_Recreation_IPAQ / MPA_Work_IPAQ, physical activity duration at moderate intensity (3–6 METs) based on the International Physical Activity Questionnaire; MPA_Recreation_SWMA / MPA_Work_SWMA, physical activity duration at moderate intensity (3–6 METs) measured by the SenseWear Mini armband. * Intergroup comparisons (low-intensity vs. moderate-intensity vs. high-intensity group) revealed highly significant differences (p<0.001).

Fig 2 illustrates subjective IPAQ data across occupational groups in comparison to objective SWMA data. Based on the IPAQ, MPA and HPA at work were again significantly higher in the high-intensity group compared to the other groups. However, HPA in recreation was reduced in the high-intensity group, while no significant difference was found with the SWMA. In total subjects, MPA at work was underreported by two-thirds using the IPAQ compared to the SWMA (51 min/day, SD 73 vs. 151 min/day, SD 131), while MPA in recreation was underreported by 55% (58 min/day, SD 46 vs. 130 min/day, SD 59). In contrast, HPA was overreported by 75% during work (14 min/day, SD 36 vs. 8 min/day, SD 14) and in recreation by 60% (16 min/day, SD 21 vs. 10 min/day, SD 11).

### Determination of physical performance criteria

In Table 2, mean values of VO_2max_ and physical workload as well as lower and upper workload limits are presented across occupational groups, stratified for gender. In total subjects, mean workload was about 23% of VO_2max_ (lower limit: 14%—upper limit: 31%). The ratio of workload to maximum work capacity was inferior in the low-intensity group (16% (10% - 21%)) compared to the moderate- (20% (11% - 29%)) and high-intensity group (32% (19% - 44%)). Moreover, men exerted a lower relative workload (19% (11% - 26%)) than women (26% (16% - 37%)), when all groups were combined.

**Table 2:** Ratio of workload to maximum work capacity according to occupational group and gender.

	VO_2max_ [METs]		Workload [METs]						
Males (n = 190)	*Mean*	*SD*	*Mean*	*%VO*_*2max*_	*SD*	*-2SD*	*%VO*_*2max*_	*+2SD*	*%VO*_*2max*_
Low-intensity group	12.8	2.3	1.7	13.3	0.3	1.1	8.6	2.3	18.0
Moderate-intensity group	12.9	2.7	2.2	17.1	0.5	1.2	9.3	3.2	24.8
High-intensity group	12.7	2.1	3.3	26.0	0.6	2.1	16.5	4.5	35.4
Females (n = 113)	*Mean*	*SD*	*Mean*	*%VO*_*2max*_	*SD*	*-2SD*	*%VO*_*2max*_	*+2SD*	*%VO*_*2max*_
Low-intensity group	9.4	2.2	1.7	18.1	0.3	1.1	11.7	2.3	24.5
Moderate-intensity group	9.4	1.8	2.2	23.4	0.5	1.2	12.8	3.2	34.0
High-intensity group	8.1	1.9	3.0	37.0	0.6	1.8	22.2	4.2	51.9

METs, metabolic equivalents; SD, standard deviation; VO_2max_, maximal oxygen uptake during 20-meter shuttle run test.

### Predictors of physical workload

Forward stepwise multiple linear regression analyses with physical workload as dependent variable are shown in Table 3. In model 1, objective SWMA parameters were included as predictors, while model 2 considered subjective IPAQ variables. The overall fit of model 1 was very high explaining 93% of variance of workload. METs increased from the low- to the moderate- and high-intensity group as shown by the positive correlations. MPA, HPA and VHPA at work were also found to be positively associated with METs. In contrast, daily working hours, age, flextime and VO_2max_ showed a negative relationship with physical workload. Based on the results of multiple linear regressions, this study has generated the following prediction equation for model 1: Workload [METs] = 2.247 + (0.005 x MPA work [min/day]) + (0.007 x HPA work [min/day]) + (0.234 x Occupational group; Low-intensity = 0, High-intensity = 1) + (0.156 x Occupational group; Low-intensity = 0, Moderate-intensity = 1)–(0.056 x Working hours [h/day]) + (0.031 x VHPA work [min/day])–(0.086 x Flextime; No = 0, Yes = 1)–(0.003 x Age [yrs])–(0.010 x VO_2max_ [METs]).

**Table 3:** Forward stepwise multiple linear regressions with workload [METs] as dependent variable.

**Objective SenseWear Mini armband data**				
*Model 1*: *n = 297*, *adjusted R*^*2*^ *= 0*.*93*	*B*	*SE B*	*ß*	*p-value*
*Constant*	*2*.*247*	*0*.*157*		*<0*.*001*
MPA work [min/day]	0.005	0.000	0.736	<0.001
HPA work [min/day]	0.007	0.001	0.118	<0.001
Low- vs. high-intensity group	0.234	0.050	0.135	<0.001
Low- vs. moderate-intensity group	0.156	0.033	0.090	<0.001
Working hours [h/day]	-0.056	0.015	-0.057	<0.001
VHPA work [min/day]	0.031	0.010	0.049	0.002
Flextime: No vs. Yes	-0.086	0.031	-0.050	0.006
Age [yrs]	-0.003	0.001	-0.045	0.011
VO_2max_ [METs]	-0.010	0.005	-0.034	0.039
**Subjective International Physical Activity Questionnaire data**				
*Model 2*: *n = 296*, *adjusted R*^*2*^ *= 0*.*74*	*B*	*SE B*	*ß*	*p value*
*Constant*	*4*.*395*	*0*.*439*		*<0*.*001*
Low- vs. high-intensity group	1.114	0.083	0.650	<0.001
Low- vs. moderate-intensity group	0.326	0.065	0.191	<0.001
BMI [kg/m^2^]	-0.052	0.008	-0.221	<0.001
MPA work [min/day]	0.001	0.000	0.136	<0.001
Flextime: No vs. Yes	-0.208	0.060	-0.123	0.001
Age [yrs]	-0.008	0.002	-0.126	0.001
Gender: Male vs. Female	-0.263	0.082	-0.158	0.002
Working hours [h/day]	-0.063	0.029	-0.065	0.032
VO_2max_ [METs]	-0.029	0.014	-0.097	0.034
HPA work [min/day]	0.002	0.001	0.070	0.041

B, unstandardized regression coefficient; ß, standardized beta coefficient; BMI, body mass index; METs, metabolic equivalents; MPA / HPA / VHPA, physical activity duration at moderate (3–6 METs) / high (6–9 METs) / very high (≥9 METs) intensity; SE, standard error; VO_2max_, maximal oxygen uptake during 20-meter shuttle run test.

The adjusted R^2^ of model 2 was slightly lower but still high with 0.74. The displayed correlations were similar to model 1 with the exception of BMI and gender, which now revealed a significant negative association with workload.

*Model 1* included predictors were Gender (Male vs. Female), Age, BMI, VO_2max_, Occupational group (Low-intensity vs. moderate-intensity group, low-intensity vs. high-intensity group), Daily working hours, Daily sleeping hours, Flextime (No vs. Yes), Shift work (No vs. Yes), Weekend work (No vs. Yes), Min/day of MPA, HPA, VHPA at work (measured with the SenseWear Mini armband).

*Model 2* included predictors were Gender (Male vs. Female), Age, BMI, VO_2max_, Occupational group (Low-intensity vs. moderate-intensity group, low-intensity vs. high-intensity group), Daily working hours, Daily sleeping hours, Flextime (No vs. Yes), Shift work (No vs. Yes), Weekend work (No vs. Yes), Min/day of MPA, HPA at work (assessed by the International Physical Activity Questionnaire).

## Discussion

This cross-sectional study found that the high-intensity group including manual labourers, agricultural workers and craftsmen showed a higher proportion of MPA, HPA, VHPA and steps, as well as EE and METs measured by the SWMA on workdays during work-time than the other occupational groups. VO_2max_ was also greater in this group, but did not differ when stratified for gender. In contrast, during leisure-time on workdays, VHPA and steps were reduced in the high-intensity group compared to the low- and moderate-intensity group. No significant differences in physical activity between the groups were found on non-working days (except EE). In total subjects, mean workload as determined by METs was about 23% of VO_2max_ (lower limit: 14%—upper limit: 31%). The ratio of workload to maximum work capacity increased from the low- to the moderate- and high intensity group. Moreover, the relative workload exerted by males was lower than by females due to their higher VO_2max_. Furthermore, higher-intensity groups, MPA, HPA and VHPA at work were identified as positive predictors of physical workload, while daily working hours, age, flextime and VO_2max_ showed a negative association. Multiple linear regressions including subjective activity variables revealed similar correlations as with objective parameters, but presented a slightly lower adjusted R^2^. However, when directly comparing subjective and objective activity data, MPA during work and recreation were underreported using the IPAQ, whereas work and non-work related HPA were overreported.

### Physical activity data across occupational groups

As the analyses show, occupational groups differed considerably in physical activity and EE. The present findings are similar to those of previous studies using pedometers or accelerometers. Steele & Mummery [7] reported in Australian workers a gradation in step counts during work-time from professionals to white-collar and blue-collar workers. Mean step counts were lower by 1000–2000 steps in each group than in this study. However, they used a spring-levered pedometer that is known to be compromised in accuracy at slow walking speeds [27]. Similarly, a representative sample of Swiss workers indicated that on workdays fewer steps were accumulated in sitting occupations compared to standing occupations and physically active jobs [28]. Miller & Brown [8] also detected reduced step counts on weekdays in professionals compared to technical and blue-collar workers. Consistent with the present findings, no significant differences were found on weekend days. The lack of difference in physical activity outside work was confirmed by Tigbe *et al*. [29]. Previous studies showed in sedentary occupations that leisure-time included more physical activity than work-time [9, 30], which is in line with the present results. However, this study showed that leisure-time activity was not increased in the low-intensity group compared to the other groups. Therefore, when total activity was considered, employees of the moderate- and high-intensity group accumulated more physical activity. This suggests that subjects in jobs with low physical demands do not fully compensate for their inactivity at work during leisure-time.

Regarding aerobic capacity, VO_2max_ values were increased in the high-intensity group compared to the other groups, but did not differ when stratified for gender. This might be explained by the fact that 95% of subjects in the high-intensity group were men. Therefore, it is likely that this group had an increased aerobic capacity because of the large proportion of males, whose mean VO_2max_ was considerably higher than those of females. This could also be the reason for the higher EE on non-working days, while the other activity parameters did not differ significantly. Men in general have more skeletal muscle mass in comparison to women in both absolute terms and relative to body mass, which results in an increased EE [31].

### Determination of physical performance criteria

Based on the observations, this study could confirm previous findings expressing physical workload as percentage of VO_2max_. For example, Jorgensen *et al*. [32] found that the upper limit for an eight-hour workday of mixed physical work was 30–35% of VO_2max_, which is consistent with the present results (31%). However, they did not account for job-dependent differences. This investigation found that the relative workload was 1.5 times and twice as high in the high-intensity group (44%) compared to the moderate- (29%) and low-intensity (21%) group. Another study suggested that the overall workload limit for jobs with high physical demands might be within the range of 33–50% of VO_2max_ [33]. While this study did confirm these values, it showed that women had a considerably higher limit (52%) than men (35%) due to their lower VO_2max_. These differences in relative workload need to be accounted for in clinical practice with regard to a safe return to work, particularly for the high-intensity group.

### Predictors of physical workload

This is the first study analysing predictors of physical workload objectively measured by the SWMA. Evidently, workload increased from the low- to the moderate- and high-intensity group. More MPA, HPA and VHPA during work also increased workload, while flextime could decrease workload. Kelloway & Gottlieb [34] confirmed that work arrangements involving flexibility promoted women’s well-being by increasing perceived control over time and reducing perceived job overload. With increasing age and longer working hours workload needs to be reduced, which is consistent with Wu & Wang [35]. BMI and gender did not show a significant association. In this study, women presented equal absolute METs during work as men, but had a higher relative workload due to their lower aerobic capacity. VO_2max_ showed a negative association with workload. However, it was just slightly significant and therefore not one of the most important predictors. This is an interesting finding, since up to now work recommendations were primarily based on VO_2max_. To facilitate the implementation of the study results, the generated regression equation for predicting physical workload could be used to develop user-friendly calculators (e. g. mobile apps). This would enable different stakeholders (e. g. employees, employers and insurance agencies) to evaluate individuals’ physical workload in a low effort way.

For clinical practice, it might be valuable to use predictors measured by a simple instrument, rather than by the SWMA. When including self-reported IPAQ data, similar correlations were revealed as with objective data. However, the two methods for measuring physical activity showed large discrepancies. Subjective MPA data were lower than objective data and HPA were reversed. These measurement variations are in line with a Swedish study comparing the IPAQ with an accelerometer [36].

### Generalizability of results

Study subjects were equally distributed across groups with low-, moderate- and high-intensity occupational activity. The present results showed that the three groups differed significantly from each other in terms of physical workload (METs) and confirmed the applied classification. Just machine operators showed a high variance, which could be explained by the small number of subjects (n = 4, 1%). This corresponds to 4% in the Swiss working population [37]. The percentage of women in the present study was similar to data of the Swiss Labour Force in the low- and moderate-intensity group, but lower in the high-intensity group [37]. However, 78% of subjects in this group were craftsmen. When considering only craftsmen, the female percentages were comparable. Furthermore, more subjects between 18–39 years and fewer subjects between 40–65 years were included in this study [37]. This might be due to the fact that younger people were more motived to participate. Nevertheless, a healthy worker effect appears to be unlikely, since the percentage of overweight and obesity was in accordance with the prevalence in Switzerland in 2012 [38]. Moreover, mean VO_2max_ values of total, male and female subjects corresponded to a previous population-based study in US employees [39].

### Strengths and limitations

The study sample included a wide range of manual and non-manual employees and represented a typical cross-section of the Swiss working population, but the proportion of women was only 5% in the high-intensity group. In order to strengthen the observed findings, future studies need to focus on females in this subgroup. Furthermore, the measurement of physical activity and aerobic capacity was conducted with objective instruments. To the authors’ knowledge, this is the first study determining gender-related and job-specific physical performance criteria in healthy employees based on objective workload data derived in real-life workplaces. These physical performance criteria build a good basis for future investigations, but need to be validated for other populations. The two different methods for assessing physical activity indicate a substantial discrepancy between subjective and objective measurements. The SWMA promises an accurate assessment of physical activity under non-ambulatory conditions [11]. The inclusion of thermal- and perspiration-related sensors allows detecting subtle increases in physical activity associated with low intensities. Furthermore, this device ensures a sensitive determination of acceleration provoked by muscle power or externally by a vehicle or gravitation [11]. The recording of non-wearing, resting and sleep time also allow for more confidence in data consistency. However, the SWMA has been shown to underestimate activities at high intensities and those involving purely lower extremities, such as cycling, because of its wearing position on the upper arm [11, 40]. In addition, it is not waterproof and lacks to detect water-based activities. A strength of the IPAQ is its ability to assess various dimensions of physical activity, such as duration, frequency, intensity and different domains [20]. The IPAQ is suitable for the implementation in large populations, because it is cost effective and simple in use. However, there is evidence that subjects may find it difficult to differentiate between moderate and vigorous intensity and to identify the actual time spent in these activities [41]. Therefore, objective measurement methods, such as the SWMA, may be preferably used to determine detailed activity profiles across occupational groups.

### Clinical implications

This study provides objective information about employees’ work capacity and physical work requirements of different occupational groups. Based on the determined physical performance criteria, it can be evaluated whether somebody is able to resume his/her previous work after phases of sick leave. If a patient’s work ability in comparison to population-based values is sufficient to meet the minimum work requirements (lower workload limit) of his/her corresponding job group, then the patient is likely to return to work successfully. For example, a male patient previously working in the high-intensity group would like to go back to his former job after illness. He performed a 20-meter shuttle run test and achieved a VO_2max_ of 8 METs. This value divided by the mean VO_2max_ of the high-intensity group (12.7 METs) and multiplied by the corresponding mean METs (3.3 METs) results in 2.08 METs. Comparing this value to the lower limit of the high-intensity group (2.1 METs) suggests that this patient is borderline for resuming his work and his job profile may need to be adjusted. This example elucidates how data from this study may help to improve intervention strategies and clinicians’ return-to-work recommendations. An optimized reintegration process may reduce future loss of working hours and related health care costs.

## Conclusions

In a representative sample of a working population, this study found that subjects in jobs with high physical demands had increased activity levels on workdays, while physical activity on non-working days did not differ across occupational groups. Individuals in sedentary occupations did not appear to fully compensate for their inactivity at work during leisure-time. VO_2max_ was considerably higher in men compared to women, but did not differ across groups when stratified for gender. Discrepancies between subjectively rated and objectively measured activity data recommend using objective methods for accurately determining activity profiles across occupational groups. While this study did confirm that the average workload limit is one third of VO_2max_, it showed that the average is misrepresenting the actual physical work demands of specific occupational groups, and that it does not account for gender-related differences in relative workload. The determined job- and gender-specific physical performance criteria may help to develop future guidelines for a safe return to work. Results of multiple linear regressions suggest considering various personal and job-related factors for evaluating physical workload, besides VO_2max_. In a further step, the generated regression equation may be used to develop simple tools for determining individuals’ workload, such as calculators or mobile apps.

## Supporting Information

S1 Dataset Excel file of personal and job-related factors. (XLSX)

S2 Dataset Excel file of body measurements and 20-meter shuttle run. (XLSX)

S3 Dataset Excel file of SenseWear Mini armband activity parameters. (XLSX)

S4 Dataset Excel file of International Physical Activity Questionnaire variables. (XLSX)

S1 Protocol Study protocol approved by the local ethics committee. (DOC)

S1 Table Personal and job-related factors across occupational groups. (DOCX)

S2 Table Aerobic capacity and SenseWear activity data across occupational groups in men (n = 190). (DOCX)

S3 Table Aerobic capacity and SenseWear activity data across occupational groups in women (n = 113). (DOCX)
