# Supplementary material for: Physical Workload and Work Capacity across Occupational Groups
Source: PLoS One. 2016 May 2;11(5):e0154073. doi: 10.1371/journal.pone.0154073 (PMC4852946; doi:10.1371/journal.pone.0154073)
Supplement: S1 Protocol — (DOC) [file pone.0154073.s005.doc]

**Research Protocol**

**Title:**

**Physical activity and energy expenditure across occupational categories**

Principal Investigator

Prof. Jörg D. Leuppi, MD PhD, Deputy Head of Internal Medicine, University Hospital Basel, Petersgraben 4, 4031 Basel

Signature: Date: _____________________

On site collaborators

Stefanie Zogg, MSc ^1,2^

Selina Dürr, MSc ^1,2^

Nebal Abu Hussein ^1^

Sabrina Maier ^1^

Prof. Kurt Murer ^2^

David Miedinger, M.D., PhD ^1,3^

^1^ Clinic of Internal Medicine, University Hospital Basel, Switzerland

^2^ Institute of Human Movement Sciences and Sport, ETH Zurich, Switzerland

^3^ Department of Occupational Medicine, Swiss National Accident Insurance Fund (Suva) Lucerne, Switzerland

**Contents**

[1 Introduction 4](#__RefHeading___Toc340680554)

[1.1 Background 4](#__RefHeading___Toc340680555)

[1.2 Specific aims 5](#__RefHeading___Toc340680556)

[1.3 Hypotheses 5](#__RefHeading___Toc340680557)

[1.3.1 . 5](#__RefHeading___Toc340680558)

[1.3.2 . 5](#__RefHeading___Toc340680559)

[1.3.3 . 5](#__RefHeading___Toc340680560)

[1.4 Potential significance 6](#__RefHeading___Toc340680561)

[2 Methods 6](#__RefHeading___Toc340680563)

[2.1 Study design 6](#__RefHeading___Toc340680564)

[2.1.1 Primary and secondary outcomes 6](#__RefHeading___Toc340680565)

[2.1.2 Study procedures 6](#__RefHeading___Toc340680567)

[2.1.3 Flowchart 7](#__RefHeading___Toc340680568)

[2.2 Selection of study participants 8](#__RefHeading___Toc340680569)

[2.2.1 Recruitment 8](#__RefHeading___Toc340680570)

[2.2.2 Inclusion criteria 8](#__RefHeading___Toc340680571)

[2.2.3 Exclusion criteria 8](#__RefHeading___Toc340680572)

[2.3 Instruments 9](#__RefHeading___Toc340680573)

[2.3.1 Personal and job related factors 9](#__RefHeading___Toc340680574)

[2.3.2 Classification of occupations 9](#__RefHeading___Toc340680575)

[2.3.3 International Physical Activity Questionnaire 10](#__RefHeading___Toc340680576)

[2.3.4 SF-36 10](#__RefHeading___Toc340680577)

[2.3.5 Visual Analogue Scale 11](#__RefHeading___Toc340680578)

[2.3.6 Borg Scale 11](#__RefHeading___Toc340680579)

[2.4 Measurements 12](#__RefHeading___Toc340680580)

[2.4.1 Anthropometric measurements 12](#__RefHeading___Toc340680581)

[2.4.2 20 meter shuttle run 12](#__RefHeading___Toc340680582)

[2.4.3 SenseWear Mini armband 13](#__RefHeading___Toc340680583)

[2.5 Data collection, management and retention 15](#__RefHeading___Toc340680584)

[2.6 Data analysis 15](#__RefHeading___Toc340680585)

[2.6.1 Primary endpoints 15](#__RefHeading___Toc340680586)

[2.6.2 Secondary endpoints 16](#__RefHeading___Toc340680587)

[2.6.3 Statistical analysis 16](#__RefHeading___Toc340680588)

[2.6.4 Sample-size calculation 17](#__RefHeading___Toc340680589)

[2.7 Duties of the investigator 17](#__RefHeading___Toc340680590)

[2.8 Ethical consideration 17](#__RefHeading___Toc340680591)

[2.8.1 Informed consent of study participants 17](#__RefHeading___Toc340680592)

[2.8.2 Risks and benefits for study participants 18](#__RefHeading___Toc340680593)

[2.9 Publication and authorship 18](#__RefHeading___Toc340680594)

[2.10 References 19](#__RefHeading___Toc340680595)

# Introduction

## 1.1 Background

Regarding occupational rehabilitation, a balance between workload and individual’s work capacity is required for returning to work successfully after an injury or illness. Therefore, it is of great value to evaluate a wide range of physical work requirements and to assess employee’s work capacity across occupational categories. Although several approaches have been made in this regard, there are few objective data available so far and no established thresholds exist. One reason could be that objectifying workload and work capacity is methodically challenging because of their multidimensional nature .

Physical activity is defined as any bodily movement produced by skeletal muscles resulting in energy expenditure *.* In classical work physiology, energy expenditure was therefore considered as basis for assessing the amount of workload *.* However, the energy cost of physical activity may not necessarily be equivalent to body movement . For that reason, modern concepts include also other aspects reflecting the severity of manual work. The ‘Dictionary of Occupational Titles’ (DOT), developed by the U.S. government, classifies professions into five categories (sedentary, light, medium, heavy, very heavy) based on the amount of energy expended as well as on the intensity and duration of lifting or carrying during work . Though, the DOT classification has not been based on quantitative work-related analyses, but rather on consensus meetings of experts. Furthermore, its validity has not been established.

Work capacity can be assessed using functional capacity evaluations (FCEs), which measure the ability of subjects to perform specific activities . Worldwide, there are various FCE protocols available being job specific , pathology specific or of a more general type. Soer et al. applied an evaluation system consisting of 12 work-related tests to establish functional capacity in healthy employees . The assessment included various lifting and energetic exercises as well as coordination tasks. From the test results, normative FCE values were acquired for each DOT-category, which can be compared to patient data in order to make work recommendations.

However, since validity of the DOT has not been proved, further analysis concerning workload assessment is required. Moreover, the application of such complex FCE tools is related to high expenses and efforts and may therefore not be appropriate in a general clinical or field context. Especially in large populations, implementing highly accurate instruments is difficult due to high costs and poor practicability .

For these reasons, the present study will focus on workload, rather than work capacity, by investigating a wide range of physical work requirements across occupational categories. Since physical activity is characterized by various dimensions, such as duration and intensity , these factors will be considered for a comprehensive analysis. However, not only absolute values, but also the distribution of activity pattern may play a role in this context and will therefore be evaluated.

## 1.2 Specific aims

The primary aim of this study is to investigate healthy employees in Switzerland regarding active energy expenditure, physical activity duration at different intensities and the distribution of medium- and high-intensity activity periods of more than ten minutes during a normal working day with focus on differences between occupational categories. Furthermore, the association between physical activity and exercise capacity will be examined.

## 1.3 Hypotheses

### Individuals practising heavy work are more physically active and consume more energy than those with light work.

- - 1. Exercise capacity (VO_2max_) correlates positively with work and non-work related physical activity.

### 1.3.3 Better trained subjects (with a higher aerobic capacity) show longer periods of medium- and high-intensity activity than untrained individuals.

## 1.4 Potential significance

This study is expected to provide detailed information about physical work requirements and performance criteria of different job categories. This may be valuable regarding treatment and professional reintegration after an injury or illness, since up to now no established thresholds exist. Objective workload data might improve clinicians’ recommendations for a successful return to work.

# Methods

## 2.1 Study design

### 2.1.1 Primary and secondary outcomes

## Our primary objective is to analyze work related physical activity parameters measured by the SenseWear Mini armband across occupational categories. Furthermore, exercise capacity, perceived work intensity, activity behaviour in recreation, quality of life, body composition and several personal and job factors will be investigated as secondary outcomes.

### 2.1.2 Study procedures

In this cross-sectional study, we plan to recruit healthy full-time employed adults from different occupational fields in the area of Basel, Switzerland. It is intended to start with a pilot study in January 2013, in which a total of 15 persons will be examined.

Recruitment will initiate in April 2013 and will end as soon as 300 individuals are included in the study (at the latest in March 2014).

Potential subjects will be contacted and asked for study participation by phone or by mail. Willing individuals meeting the inclusion criteria will fill out the informed consent form before starting the measurement procedure.

At the first study visit, body measurements will be carried out such as height, weight and waist circumference (WC) and body mass index (BMI) will be calculated. In addition, a variety of personal and job related factors will be recorded. Furthermore, subjects will perform a 20 meter shuttle run in order to measure exercise capacity.

During the subsequent week, participants will be instructed to document their daily work activities and to rate their work related physical exertion twice per day using the Borg Scale. Moreover, by wearing the SenseWear Mini armband on seven consecutive days, physical activity and energy expenditure will be objectified.

One week later at the second study visit, all subjects will complete several self-administered questionnaires concerning work and non-work related physical activity behaviour (IPAQ), health-related quality of life (SF-36) and self-assessment of professional activity and its intensity (VAS). In addition, open questions will be asked to each participant about past-week physical exertion in comparison to a normal week.

The time required for the two study visits taking place at the individual’s workstation, will be around 90 minutes per person. In addition, every participant will wear the activity monitor on seven consecutive days.

Termination criteria for the measurements are skin irritations caused by the SenseWear Mini armband and exertional dyspnea during 20 meter shuttle run.

### 2.1.3 Flowchart

|  | Day **1** | **2** | **3** | **4** | **5** | **6** | **7** | Day **8** |
| --- | --- | --- | --- | --- | --- | --- | --- | --- |
| **Examination** | Body measurements  (Height, weight, WC)  Interview  (Personal and job related factors)  20 meter shuttle run  Instructions for SenseWear Mini Armband | * Wearing period of SenseWear Mini Armband  ° Borg Scale  ^#^ Documentation of  work activities |  |  |  |  |  | Questionnaires  (IPAQ, SF-36, VAS,  open questions)  Collection of SenseWear Mini Armband |
| **Location** | At work | * At work and at home  ^°, #^ At work |  |  |  |  |  | At work |
| **Time interval** | 60 min | * 7 consecutive days, 23h/day  ° 2x/day (midday, evening)  ^#^ 1x/day (evening) |  |  |  |  |  | 30 min |

## 2.2 Selection of study participants

### 2.2.1 Recruitment

In this study, it is intended to recruit 300 subjects from different occupational groups with low, middle and high occupational activity, which are defined in a later section (see chapter 2.3.2). Based on this classification, companies will be selected. In each occupational group, 100 participants will be enrolled. Furthermore, homogeneity of age and gender within each group will be aimed at recruiting.

First, we will contact the management of potential enterprises in order to present our project. If they are interested in collaboration, we will ask for a list of employees, who will then be addressed by a member of our research team. In a further step, individuals will be informed and asked for study participation by phone or by mail. Furthermore, it is planned to distribute study flyers in the companies, so that interested employees have the opportunity to contact us directly. In both situations, the recruitment and information process will be done externally. Thus, negative impact of the employer can be avoided and the selection of study participants will occur randomly. However, the employer’s agreement is essential for the conduction of the study.

### 2.2.2 Inclusion criteria

Healthy full-time employed individuals between the age of 18 and 65, who have a sufficient knowledge of the German language in order to fill in the self-administered questionnaires by themselves.

### 2.2.3 Exclusion criteria

Subjects not giving consent for participation as well as those with movement restrictions, various diseases and accidents within the last three months that affect productivity at the workplace, cannot take part in this study. Furthermore, shift workers and individuals, who are exposed to high-risk explosive environments, will be excluded from participation.

## Instruments

### 2.3.1 Personal and job related factors

For each subject, gender *(male / female)* and employment *(temporary / permanent)* will be assessed as binary variables. Nationality *(Swiss / EU / other)*, marital status *(single / married / divorced)*, and smoking status *(yes / no / never)* will be recorded with three categories, while alcohol consumption *(never / once to several times per month / once to several times per week / once per day),* German skills *(very good / good / medium / bad / very bad)*, highest education *(compulsory school / apprenticeship / higher vocational school / diploma or maturity / university)* and working time model *(flexitime / fix time / shift work / night work / weekend work / short work)* will be evaluated with four, five and six categories, respectively. Age, native language, profession, avocation, medication, illnesses and accidents and working hours will be asked as open questions. Furthermore, to get an overview of the individual work activity, participants will be asked to keep a daily list of the main completed tasks including time information in accordance with the questionnaire regarding professional integration and pension .

### 2.3.2 Classification of occupations

The reported professions will be classified into 9 categories based on the International Standard Classification of Occupations ISCO-88  *(1. Managers and administrators, 2. Professionals, 3. Technicians and associate professionals, 4. Clerks, 5. Service workers, 6. Agricultural and fishery workers, 7. Construction and related workers, 8. Plant and machine operators, 9. Elementary workers)*. These 9 categories will then be collapsed into 3 groups with low, middle and high occupational activity according to Suva experts in the field of ergonomics (*1^st^ group*: Categories 1 (Managers and administrators) and 2 (Professionals); *2^nd^ group*: Categories 3 (Technicians and associate professionals), 4 (Clerks) and 5 (Service workers); *3^rd^ group*: Categories 6 (Agricultural and fishery workers), 7 (Construction and related workers), 8 (Plant and machine operators) and 9 (Elementary workers). Reliability of this stratification has been previously shown .

### 2.3.3 International Physical Activity Questionnaire

The International Physical Activity Questionnaire (IPAQ) represents a convenient and simple instrument for measuring health enhancing physical activity at the population level. The self-administered long version of the IPAQ, being freely available in internet , will be used in the present study. It includes 26 questions and assesses past-week frequency (days) and duration (time per day) of physical activity within the domains of work, leisure-time, transport and domestic & garden. Moreover, each domain consists of walking, moderate and vigorous activities. In addition, daily sitting time at weekdays and weekend days is recorded.

According to the IPAQ guidelines for data analysis , continuous scores in form of MET-minutes per week will be calculated for each domain. MET-minutes per week, defined as energy costs of physical activity, will be computed by multiplying the intensity (specific MET value) of an activity with its frequency (days) and duration (minutes). The corresponding MET values are set by the IPAQ guidelines based on the compendium of physical activity .

According to numerous validity and reliability tests carried out in twelve different countries, the IPAQ is considered as an internationally valid assessment tool with acceptable measurement properties . The long version of the IPAQ was translated into German as part of a dissertation and was validated in an Austrian sample . It could be shown that it is suitable for research purposes with regard to physical activity detection .

### 2.3.4 SF-36

The SF-36 is a general quality of life questionnaire consisting of 36 items, which are formatted as binary questions or as semantic six-point differential scales. It refers to the past four weeks and includes nine content areas concerning vitality, general health perception, physical functioning, social functioning, role limitations (emotional/physical problems), pain, mental health and health change . The SF-36 is considered to be the gold standard for measuring functional status and health-related quality of life and is therefore the most used questionnaire in this context .

Since physical and mental disorders as well as pain may influence activity behaviour, the SF-36 will be used in the present study to adjust for potential confounders.

### 2.3.5 Visual Analogue Scale

The Visual Analogue Scale (VAS) is a measuring instrument for subjective characteristics that cannot be directly measured. Individuals express their response to a VAS item by marking a position along a continuous line between two end-points.

The indicated locus will then be quantified by a defined scale . In epidemiologic and clinical research, the most common VAS is a straight horizontal line of 100 mm length with its ends representing extreme limits of the measured parameter (worst - best) . The VAS score is determined by measuring the distance in mm from the left end of the line to the point marked by the participant. Validity and reliability of VAS have been scientifically tested for a variety of parameters, such as acute and chronic pain .

In this study, individual’s perception of past-week work and non-work related physical exertion and effort will be assessed by 100mm VASs. This may be valuable to evaluate whether the investigated period (exposure time) is representative and reflects a normal week.

### 2.3.6 Borg Scale

The Borg Scale represents a simple method to rate perceived exertion and can be compared to other scales, such as VAS. It is used for various purposes, such as measuring patient’s exertion during a performance test or assessing the intensity of training and competition in athletes .

The original scale developed by Borg ranging from 6 (no exertion at all) to 20 (maximal exertion) was updated and transformed into a combined category-ratio scale CR10 with the ratings 0 (nothing at all) to 10 (very very hard) .

The latter will be used in the present study. Subjects will be instructed to rate their work related physical exertion twice per day, at the end of the morning and in the late afternoon. The indicated Borg value should reflect how heavy and strenuous work feels to them considering all sensations of physical stress, effort and fatigue.

## Measurements

### 2.4.1 Anthropometric measurements

Anthropometric measurements include body height, body weight and waist circumference. Body height will be assessed without shoes by a medical measuring stick to the nearest mm. The measurement of body weight will be performed on subjects in light clothing without shoes by a medical scale with an accuracy of 0.1 kg.

Waist circumference will be determined midway between the lowest rib and the iliac crest according to the Swiss Heart Foundation using a medical measuring tape with a precision of 0.1 cm. The measurement will be carried out on standing subjects after a moderate expiration.

### 2.4.2 20 meter shuttle run

The multistage 20 meter shuttle run is a common endurance fitness test to evaluate the maximal aerobic power of healthy adults . It is simple in use and economical and large groups can be tested at once.

This test will be conducted on a flat, non-slip surface and participants will be instructed to run back and forth between two lines, which are 20 meters apart (Figure 1). Running velocity is determined by intervals between two audio signals emitted by a pre-recorded tape. Subjects have to touch the 20 meter lines simultaneously with the sound of these signals. The initial running velocity is 8.0 km/h. Every minute (stage), the intervals are shortened and speed is increased by 0.5 km/h. The test ends when the subject gives up or when he can no longer keep the pace and does not reach the line in time (> 3 metre away from the 20 meter line) twice in a row.

**Figure 1:** Schematic illustration of the 20 meter shuttle run

The test result corresponds to the number of reached stages. According to a given table , this score is used to predict maximal oxygen uptake (VO_2max_), which can be compared to age-dependent normative data for males and females.

Validity of the 1 minute stage version of the 20 meter shuttle run to predict VO_2max_ in adults was established by Léger et al. , who compared the maximal shuttle run speed to VO_2max_ attained during a multistage treadmill test.

In addition, blood pressure and oxygen saturation will be assessed prior to testing in order to detect potential contraindications. Resting pulse and recovery pulse (2 minutes after test) will be measured and heart rate will be continuously recorded during test. Furthermore, participants will be instructed to rate their perceived physical exertion before and after test using the Borg scale.

### 2.4.3 SenseWear Mini armband

The SenseWear Mini armband is a small, lightweight and wireless multisensory activity monitor developed by Bodymedia (Pittsburgh, Pennsylvania, USA), which integrates motion data from a three-axis accelerometer along with several other physiological sensors such as heat flux, skin temperature and galvanic skin response. Validity was established by Johannsen et al. comparing energy expenditure estimates of the SenseWear Mini armband against the criterion method ‘Doubly-Labelled-Water’ in healthy adults.

The subjects will be instructed to wear the SenseWear Mini armband on the upper left arm (triceps area) for 7 consecutive days, including while sleeping, with the exception of the time spent on personal hygiene (23 hours per day). Reliability of this assessment period has been previously shown .

For recording non-wearing periods, such as water activities and showers, participants will be asked to keep a diary. The physiological data collected by the armband’s sensors will be processed by specific algorithms (professional software V.7.0, algorithm V.2.2.4) to calculate participants’ daily active energy expenditure, physical activity duration at different intensities and number of steps. Gender, age, height, weight, smoking status and handedness will also be considered in these calculations.

The output of a healthy person created by the SenseWear Mini software is presented in Figure 2. The variables of interest are marked with a circle. Furthermore, it can be seen that that the Mini armband was worn on 5 consecutive days, while the large numbers represent the average over these days.

**Figure 2:** SenseWear Mini output of a healthy person.

## 2.5 Data collection, management and retention

A member of the study team will contact employees from selected companies around Basel. Participation is voluntary and can be revoked at any time. There is no disadvantage for individuals that do not take part in the study.

To ensure a standardized process, measurements will be carried out always by the same study nurses trained for this purpose. For data collection and management, data will be anonymized and participants will be identified by a study number. Test results will be transmitted to the participants after all investigations have been performed.

All study related records and informed consent documents will be retained in the Clinic of Internal Medicine at the University Hospital of Basel as long as required by the applicable Swiss regulatory requirements (10 Years). Moreover, monitoring visits, audits and regulatory inspections will be granted direct access to the obtained data.

## 2.6 Data analysis

For the specific analysis of occupational activity parameters, working hours will be defined as effective time spent at work, whereas the way to work and breaks in between will not be taken into account. Participants will be instructed to keep a diary for documenting working time.

### Primary endpoints

Primary endpoints will include the following measurements of the SenseWear Mini armband during working time:

1. - Average daily active energy expenditure [cal]
2. - Average daily physical activity duration at different intensities [min]

(Low (<3.0 METs), middle (3.0-6.0 METs), high (6.0-9.0 METs) and very high (>9.0 METs))

1. - Average daily number of steps
2. - Graphs with plotted distribution of physical activity periods

### 2.6.2 Secondary endpoints

1. - IPAQ: Total and subtotal domain specific scores (work, leisure-time, transport,

domestic & garden [MET-min/week] and sitting time [min/week])

1. - SF-36: Total and subtotal domain specific scores (9 domains)
2. - VAS: Scores of perceived work and leisure-time physical exertion and effort
3. - Borg scale: Values of rated physical exertion at work (midday and evening)
4. - Results of the 20 meter shuttle run: predicted VO_2max_ [METs]
5. - Results of the SenseWear Mini armband in recreation: activity and energy scores

as listed above

### 2.6.3 Statistical analysis

Statistical analyses will be done using the software R (version 2.15.1) and IBM SPSS Statistics (version 19.0). Statistical significance will be acquired if a p value of less than 0.05 is attained.

Data will be presented by descriptive statistics within occupational groups. Cross tables are reported as counts and percentages. Ordinal variables are reported as median and Interquartile range (IQR). Metric variables are reported as mean and standard deviation (SD).

To evaluate primary endpoints, linear regressions will be performed. Independent variable is “occupational group”, dependent variables are “average daily active energy expenditure”, “average daily physical activity duration” and “average daily number of steps”. Optionally, personal and job related factors can be included in the regression analyses as covariates. If appropriate, data will be log-transformed. Regression results will be presented as differences of means between “occupational categories” with corresponding 95% confidence intervals (C.I.) and p-values. In case of logarithmic distributed data, geometric mean ratios will be presented.

To evaluate secondary endpoints, linear regressions will be conducted. In case of ordinal variables, non-parametric methods will be applied.

Furthermore, the mean and individual time courses of physical activity periods will be plotted for all primary and secondary variables.

### 2.6.4 Sample-size calculation

Since “average daily active energy expenditure” measured by the SenseWear Mini armband represents a valid and reliable parameter, it is considered to be the most important primary endpoint of the present investigation. For this reason, only this variable is subject to a power calculation. In a previous study, the SD of “average daily active energy expenditure” during a normal work shift was found to be 730 kcal . The expected mean difference between the three occupational groups will be around 500 kcal. Assuming a sample size of 100 subjects in each occupational group, there is a power of >90% to detect a mean difference of 500 kcal between any of these groups. This calculation is based on the assumption of a within group SD of 730 kcal and on a two-sided significance level of 5%.

## 2.7 Duties of the investigator

This study will be conducted in compliance with the approved research protocol. In case of any considerable deviations from study protocol, the investigator will send an amendment for further approval to the corresponding Ethical Committee.

Furthermore, the investigator declares that the insurance coverage for total study length will be assured before enrolling subjects into the present investigation. However, study participants will not receive financial compensation for their participation.

## 2.8 Ethical consideration

### 2.8.1 Informed consent of study participants

Prior to study entry, participants will be given an information sheet about the aim, procedures and potential risks of the study. Only after signing the informed consent form, subjects will be submitted to any study procedures. The documents used in this study will be submitted with the protocol to the Ethical Committee for approval.

### 2.8.2 Risks and benefits for study participants

In general, the present investigations are low-risk. However, by wearing the SenseWear Mini armband, allergic skin irritations may occur at the point of contact. Moreover, sleep quality might be slightly impaired during the measurement period of one week, since the armband will be continuously worn, including while sleeping. In addition, the performance of the 20 meter shuttle run representing a maximal exercise test, could lead to exertional dyspnea and a decrease of oxygen saturation that require test termination. Other risks of this study are related to privacy issues. Therefore, subjects will be anonymized and data will be entered only into password-protected computers at the University Hospital. Furthermore, measurements are intended to be conducted at the individual’s workstation in order to minimize loss of working time.

On the other hand, participants may profit from data about daily energy expenditure, number of steps and physical activity duration at different intensities. Since these parameters will be assessed during work and recreation, subjects will receive detailed information about physical work requirements as well as active leisure-time behaviour. Furthermore, results on exercise capacity will be provided. The findings of this study, concerning performance criteria of different job categories, may also be beneficial for other individuals, who need to be reintegrated into daily work after an accident or illness.

## 2.9 Publication and authorship

It is planned to publish the present study in a work, health and activity related journal, as for example ‘Journal of Occupational Rehabilitation’, ‘Journal of Occupational and Environmental Medicine’, ‘Occupational and Environmental Health’, ‘Medicine and Science in Sports and Exercise‘ or ‘International Journal of Sports Medicine’.

A first publication will focus on the characterization of professions regarding physical activity profiles and performance criteria. These results could be implemented in the areas of prevention and occupational rehabilitation in order to evaluate individual’s job qualification as well as work ability. In a second study, the relationship between exercise capacity and work and non-work related physical activity will be investigated. These results might be valuable to assess the interaction between work load and work capacity.

The authors of the publication will be determined by Stefanie Zogg (first author) and Jörg Leuppi (principle investigator) prior to submission based on their contributions made during the project. Participants without significant involvement in all stages of the project will be listed in the Acknowledgements. Furthermore, a mention in the protocol is not necessarily equated to authorship.

## 2.10 References
