# Supplementary material for: Physical Workload and Work Capacity across Occupational Groups
Source: PLoS One. 2016 May 2;11(5):e0154073. doi: 10.1371/journal.pone.0154073 (PMC4852946; doi:10.1371/journal.pone.0154073)
Supplement: S1 Table — (DOCX) [file pone.0154073.s006.docx]

|  | Total (n=303) | | Low-intensity group (n=101) | | Moderate-intensity group (n=102) | | High-intensity group (n=100) | |  |
| --- | --- | --- | --- | --- | --- | --- | --- | --- | --- |
|  | *Mean* | *SD* | *Mean* | *SD* | *Mean* | *SD* | *Mean* | *SD* | *p-value* |
| Working hours [h/day] | 8.8 | 0.8 | 8.9 | 1.0 | 8.7 | 0.9 | 8.6 | 0.5 | 0.061 |
|  | *N* | *%* | *N* | *%* | *N* | *%* | *N* | *%* |  |
| Nationality |  |  |  |  |  |  |  |  | 0.359 |
| *Swiss* | 221 | 73 | 77 | 76 | 76 | 75 | 68 | 68 |  |
| *European* | 70 | 23 | 22 | 22 | 23 | 22 | 25 | 25 |  |
| *Other* | 12 | 4 | 2 | 2 | 3 | 3 | 7 | 7 |  |
| Marital status |  |  |  |  |  |  |  |  | **0.007** |
| *Single* | 195 | 65 | 56 | 55 | 65 | 64 | 74 | 74 |  |
| *Married* | 89 | 29 | 38 | 38 | 26 | 25 | 25 | 25 |  |
| *Divorced* | 19 | 6 | 7 | 7 | 11 | 11 | 1 | 1 |  |
| Smoking status |  |  |  |  |  |  |  |  | **0.008** |
| *Never smoker* | 179 | 59 | 66 | 65 | 61 | 60 | 52 | 52 |  |
| *Ex-smoker* | 60 | 20 | 20 | 20 | 25 | 24 | 15 | 15 |  |
| *Current smoker* | 64 | 21 | 15 | 15 | 16 | 16 | 33 | 33 |  |
| Alcohol consumption |  |  |  |  |  |  |  |  | 0.084 |
| *Never* | 46 | 15 | 9 | 9 | 22 | 22 | 15 | 15 |  |
| *1-x times per month* | 146 | 48 | 52 | 51 | 51 | 50 | 43 | 43 |  |
| *1-x times per week* | 108 | 36 | 40 | 40 | 28 | 27 | 40 | 40 |  |
| *Daily* | 3 | 1 | 0 | 0 | 1 | 1 | 2 | 2 |  |
| Highest education |  |  |  |  |  |  |  |  | **<0.001** |
| *Basic school* | 11 | 4 | 0 | 0 | 1 | 1 | 10 | 10 |  |
| *Apprenticeship* | 147 | 48 | 26 | 25 | 42 | 41 | 79 | 79 |  |
| *Vocational school* | 39 | 13 | 19 | 19 | 14 | 14 | 6 | 6 |  |
| *Maturity or diploma* | 32 | 11 | 16 | 16 | 12 | 12 | 4 | 4 |  |
| *University* | 74 | 24 | 40 | 40 | 33 | 32 | 1 | 1 |  |
| Medication | 65 | 22 | 30 | 30 | 26 | 26 | 9 | 9 | **0.001** |
| Illnesses/Accidents | 69 | 23 | 20 | 20 | 26 | 26 | 23 | 23 | 0.626 |
| Psychotherapy | 48 | 16 | 19 | 19 | 21 | 21 | 8 | 8 | **0.030** |
| Flextime | 102 | 34 | 67 | 66 | 26 | 26 | 9 | 9 | **<0.001** |
| Fixtime | 191 | 63 | 35 | 35 | 68 | 67 | 88 | 88 | **<0.001** |
| Shift work | 25 | 8 | 2 | 2 | 17 | 17 | 6 | 6 | **<0.001** |
| Weekend work | 59 | 20 | 12 | 12 | 38 | 37 | 9 | 9 | **<0.001** |

**S1 Table.** **Personal and job-related factors across occupational groups.**

SD, standard deviation; Significant p-values are highlighted in bold.
