# Supplementary material for: Physical Workload and Work Capacity across Occupational Groups
Source: PLoS One. 2016 May 2;11(5):e0154073. doi: 10.1371/journal.pone.0154073 (PMC4852946; doi:10.1371/journal.pone.0154073)
Supplement: S2 Table — (DOCX) [file pone.0154073.s007.docx]

|  |  | Low-intensity group (n=55) | | Moderate-intensity group (n=40) | | High-intensity group (n=95) | |  |
| --- | --- | --- | --- | --- | --- | --- | --- | --- |
|  |  | *Mean* | *SD* | *Mean* | *SD* | *Mean* | *SD* | *p-value* |
| VO_2max_ | [ml/kg/min] | 45 | 8 | 45 | 10 | 44 | 7 | 0.795 |
| EE | Workday [kcal] | 2534 | 366 | 3113 | 1072 | 3630 | 630 | **<0.001** |
|  | *Work-time [kcal]* | 1180 | 279 | 1421 | 366 | 2198 | 434 | **<0.001** |
|  | *Leisure-time [kcal]* | 1355 | 344 | 1692 | 1033 | 1432 | 452 | **0.032** |
|  | Non-working day [kcal] | 2303 | 533 | 2261 | 586 | 2381 | 650 | 0.785 |
| METs | Workday | 2.0 | 0.3 | 2.3 | 0.4 | 2.8 | 0.5 | **<0.001** |
|  | *Work-time* | 1.7 | 0.3 | 2.2 | 0.5 | 3.3 | 0.6 | **<0.001** |
|  | *Leisure-time* | 2.4 | 0.5 | 2.3 | 0.5 | 2.3 | 0.5 | 0.344 |
|  | Non-working day | 2.1 | 0.4 | 2.1 | 0.6 | 2.1 | 0.6 | 0.834 |
| MPA | Workday [min] | 161 | 77 | 241 | 118 | 411 | 134 | **<0.001** |
|  | *Work-time [min]* | 56 | 50 | 117 | 86 | 298 | 109 | **<0.001** |
|  | *Leisure-time [min]* | 106 | 49 | 124 | 63 | 112 | 49 | 0.423 |
|  | Non-working day [min] | 179 | 95 | 186 | 106 | 193 | 121 | 0.969 |
| HPA | Workday [min] | 11 | 12 | 17 | 20 | 29 | 22 | **<0.001** |
|  | *Work-time [min]* | 1 | 3 | 6 | 11 | 19 | 18 | **<0.001** |
|  | *Leisure-time [min]* | 10 | 11 | 11 | 12 | 10 | 11 | 0.837 |
|  | Non-working day [min] | 12 | 15 | 14 | 33 | 13 | 17 | 0.690 |
| VHPA | Workday [min] | 3 | 6 | 4 | 5 | 2 | 6 | **<0.001** |
|  | *Work-time [min]* | 0 | 0 | 0 | 2 | 0 | 2 | 0.074 |
|  | *Leisure-time [min]* | 3 | 6 | 3 | 5 | 1 | 5 | **0.001** |
|  | Non-working day [min] | 1 | 4 | 4 | 10 | 2 | 9 | 0.755 |
| Steps | Workday | 9555 | 3118 | 12245 | 3826 | 15276 | 4139 | **<0.001** |
|  | *Work-time* | 3629 | 2014 | 6071 | 2598 | 10366 | 3737 | **<0.001** |
|  | *Leisure-time* | 5926 | 3077 | 6175 | 2986 | 4910 | 2271 | **0.030** |
|  | Non-working day | 8478 | 3644 | 10164 | 5868 | 9239 | 9289 | 0.277 |

**S2 Table.** **Aerobic capacity and SenseWear activity data across occupational groups in men (n=190).**

EE, energy expenditure; METs, metabolic equivalents; MPA / HPA / VHPA, physical activity duration at moderate (3-6 METs) / high (6-9 METs) / very high (≥9 METs) intensity; SD, standard deviation; VO_2max_, maximal oxygen uptake during 20-meter shuttle run test. Significant p-values are highlighted in bold.
