# Supplementary material for: Physical Workload and Work Capacity across Occupational Groups
Source: PLoS One. 2016 May 2;11(5):e0154073. doi: 10.1371/journal.pone.0154073 (PMC4852946; doi:10.1371/journal.pone.0154073)
Supplement: S3 Table — (DOCX) [file pone.0154073.s008.docx]

|  |  | Low-intensity group (n=46) | | Moderate-intensity group (n=62) | | High-intensity group (n=5) | |  |
| --- | --- | --- | --- | --- | --- | --- | --- | --- |
|  |  | *Mean* | *SD* | *Mean* | *SD* | *Mean* | *SD* | *p-value* |
| VO_2max_ | [ml/kg/min] | 33 | 8 | 33 | 6 | 29 | 7 | 0.451 |
| EE | Workday [kcal] | 1968 | 305 | 2210 | 377 | 2294 | 276 | **0.002** |
|  | *Work-time [kcal]* | 895 | 194 | 1142 | 265 | 1379 | 155 | **<0.001** |
|  | *Leisure-time [kcal]* | 1073 | 248 | 1068 | 262 | 914 | 157 | 0.444 |
|  | Non-working day [kcal] | 1968 | 1055 | 1808 | 439 | 1508 | 361 | 0.257 |
| METs | Workday | 2.0 | 0.3 | 2.2 | 0.3 | 2.6 | 0.5 | **0.003** |
|  | *Work-time* | 1.7 | 0.3 | 2.2 | 0.5 | 3.0 | 0.6 | **<0.001** |
|  | *Leisure-time* | 2.3 | 0.4 | 2.2 | 0.3 | 2.3 | 0.5 | 0.070 |
|  | Non-working day | 2.1 | 0.4 | 2.0 | 0.4 | 2.2 | 0.7 | 0.576 |
| MPA | Workday [min] | 150 | 62 | 198 | 97 | 299 | 113 | **0.004** |
|  | *Work-time [min]* | 47 | 29 | 103 | 73 | 214 | 72 | **<0.001** |
|  | *Leisure-time [min]* | 104 | 43 | 95 | 45 | 85 | 45 | 0.302 |
|  | Non-working day [min] | 168 | 85 | 161 | 100 | 154 | 116 | 0.535 |
| HPA | Workday [min] | 8 | 8 | 8 | 14 | 21 | 25 | 0.911 |
|  | *Work-time [min]* | 1 | 1 | 2 | 3 | 19 | 24 | **0.030** |
|  | *Leisure-time [min]* | 7 | 7 | 7 | 14 | 2 | 2 | 0.138 |
|  | Non-working day [min] | 11 | 14 | 8 | 12 | 6 | 10 | 0.362 |
| VHPA | Workday [min] | 2 | 4 | 1 | 3 | 0 | 0 | 0.093 |
|  | *Work-time [min]* | 0 | 0 | 0 | 0 | 0 | 0 | 1.000 |
|  | *Leisure-time [min]* | 2 | 5 | 1 | 3 | 0 | 0 | 0.093 |
|  | Non-working day [min] | 3 | 8 | 2 | 5 | 0 | 0 | 0.529 |
| Steps | Workday | 10042 | 3103 | 11305 | 3533 | 10911 | 3291 | **0.040** |
|  | *Work-time* | 3674 | 1419 | 5665 | 2466 | 5672 | 1944 | **<0.001** |
|  | *Leisure-time* | 6368 | 2651 | 5641 | 2079 | 5239 | 1805 | 0.463 |
|  | Non-working day | 9093 | 4002 | 8624 | 3338 | 5599 | 3094 | 0.144 |

**S3 Table.** **Aerobic capacity and SenseWear activity data across occupational groups in women (n=113).**

EE, energy expenditure; METs, metabolic equivalents; MPA / HPA / VHPA, physical activity duration at moderate (3-6 METs) / high (6-9 METs) / very high (≥9 METs) intensity; SD, standard deviation; VO_2max_, maximal oxygen uptake during 20-meter shuttle run test. Significant p-values are highlighted in bold.
